# Supplementary material for: Maternal Origins and Haplotype Diversity of Seven Russian Goat Populations Based on the D-loop Sequence Variability
Source: Animals (Basel). 2020 Sep 9;10(9):1603. doi: 10.3390/ani10091603 (PMC7552281; doi:10.3390/ani10091603)
Supplement: Supplementary file 1 [file animals-10-01603-s001.zip › Supplementary Materials/Suppl_Tab_S1.pdf]

#	Breed (species)	Geographical origin	NCBI accession number	Haplogroup	Reference
1	Girgentana	Italy	KR059177	A	Colli et al., 2015
2	Mati	Albania	KR059146	A	Colli et al., 2015
3	Muzhake	Albania	KR059170	A	Colli et al., 2015
4	Swiss Alpine	Switzerland	KR059181	A	Colli et al., 2015
5	Verata	Spain	KR059168	A	Colli et al., 2015
6	Laos	Laos	AB044303	B	Mannen et al., 2001
7	Malaysia	Malaysia	KR059220	B	Colli et al., 2015
8	Bezoar goat (*Capra aegagrus*)	Iran	KR059219	B	Colli et al., 2015
9	Payoya	Spain	KR059225	C	Colli et al., 2015
10	Kyrgyzstan	Kyrgyzstan	KR059212	D	Colli et al., 2015
11	Bezoar goat (*Capra aegagrus*)	Iran	KR059210	D	Colli et al., 2015
12	Bezoar goat (*Capra aegagrus*)	Iran	KR059226	F	Colli et al., 2015
13	Kermanshah	Iran	KR059213	G	Colli et al., 2015
14	Khalkhali	Iran	KR059218	G	Colli et al., 2015
15	Bezoar goat (*Capra aegagrus*)	NA	NC_028161	G	Unpublished (Zhang)
16	Caucasian tur (*Capra caucasica*)	NA	NC_020683	Outgroup	Hassanin et al., 2012

References

Colli, L.; Lancioni, H.; Cardinali, I.; Olivieri, A.; Capodiferro, M.R.; Pellecchia, M.; Rzepus, M.; Zamani, W.; Naderi, S.; Gandini, F.; et al. Whole mitochondrial genomes unveil the impact of domestication on goat matrilineal variability. BMC Genomics 2015, 16, 1115. https://doi.org/10.1186/s12864-015-2342-2.

Mannen, H.; Nagata, Y.; Tsuji, S. Mitochondrial DNA reveal that domestic goat (Capra hircus) are genetically affected by two subspecies of bezoar (Capra aegagurus). Biochemical genetics 2001, 39(5-6), 145–154. https://doi.org/10.1023/a:1010266207735

Zhang, K.; Chen, X.; Zhao, S. Complete mitochondrial genome of the wild goat (Capra aegagrus). Unpublished

Hassanin, A.; Delsuc, F.; Ropiquet, A.; Hammer, C.; Jansen van Vuuren, B.; Matthee, C.; Ruiz-Garcia, M.; Catzeflis, F.; Areskoug, V.; Nguyen, T. T.; Couloux, A. Pattern and timing of diversification of Cetartiodactyla (Mammalia, Laurasiatheria), as revealed by a comprehensive analysis of mitochondrial genomes. Comptes rendus biologies 2012, 335(1), 32-50. https://doi.org/10.1016/j.crvi.2011.11.002
