# Supplementary figures and images for: Maternal Origins and Haplotype Diversity of Seven Russian Goat Populations Based on the D-loop Sequence Variability
Source: Animals (Basel). 2020 Sep 9;10(9):1603. doi: 10.3390/ani10091603 (PMC7552281; doi:10.3390/ani10091603)

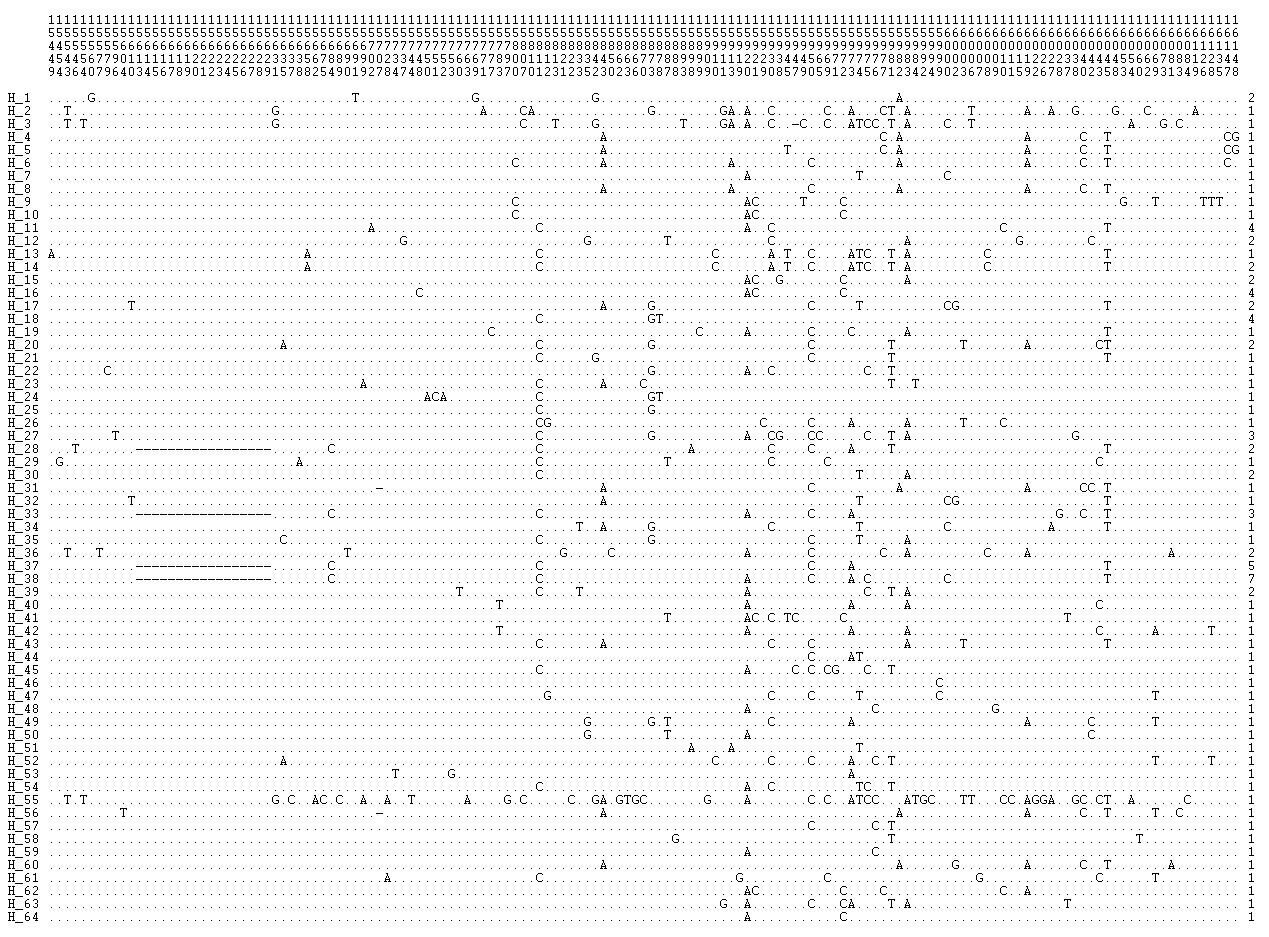

Supplement: Supplementary file 1 [file animals-10-01603-s001.zip › Supplementary Materials/Suppl_Fig_S1.png]
